# (2*E*)-2-[(4-Eth­oxy­phen­yl)imino]-1,2-di­phenylethanone

**DOI:** 10.1107/S2414314626007406

**Published:** 2026-07-28

**Authors:** Chaabane Chiter, Yamina Boudinar, Hibet Errahmane Meroua Akkache, Amel Djedouani, Salima Tabti, Erwann Jeanneau

**Affiliations:** a Laboratoire de Chimie Ingénierie Moléculaire et Nanostructures Université Ferhat Abbas Sétif 1 Sétif 19000 Algeria; b Department of Chemistry, Faculty of Sciences, University of 20 August 1955, Skikda 21000, Algeria; c Laboratory of Physico-Chemistry Research on Surfaces and Interfaces University of Skikda 21000 Algeria; d Crystallography Laboratory Department of Physics University of Constantine-1 25000 Constantine Algeria; e Ecole normale supérieure de Constantine-Assia Djebar-Université, Ali Mendjeli, Constantine 3, Constantine 25000, Algeria; f Laboratoire de produits d’origine végétale et de synthése organique ’PHYSYNOR’, Université Mentouri-Constantine 1, Constantine 25000, Algeria; g Department of Ecology and Environment, Faculty of Life and Natural Sciences of Earth and Universe Sciences, Mohamed El Bachir El Ibrahimi University, El Anasser, Bordj BouArreridj 34000, Algeria; h Laboratory of Electrochemistry and Environment, Mohamed El Bachir El Ibrahimi University, El Anasser, Bordj BouArreridj 34000, Algeria; i Centre de Diffractométrie Henri Longchambon, Université Claude Bernard Lyon 1, 5 rue de la Doua, 69100 Villeurbanne, France; Purdue University, USA

**Keywords:** crystal structure, imino-ketone, Schiff base, hydrogen bonding

## Abstract

The title compound, C_22_H_19_NO_2_, is an imino-ketone Schiff base. The configuration about the azomethine bond is *E*. The aromatic rings of the 1,2-di­phenyl­ethanone moiety are inclined to each other by 80.43 (7)°. The 4-eth­oxy­phenyl ring is inclined to the phenyl­ethanone ring by 81.91 (7)° and to the phenyl ring to which it is *trans* by 68.56 (7)°. In the crystal, four symmetry-related mol­ecules are linked by C—H⋯O hydrogen bonds to form square-like units with an *R*^3^_4_(36) ring motif, which are linked through bifurcated C—H⋯O hydrogen bonds to form sheets parallel to the (101) plane.

## Abstract 2

In the title compound, an imino-ketone Schiff base, the configuration about the azomethine bond is *E*.

## Graphical abstract

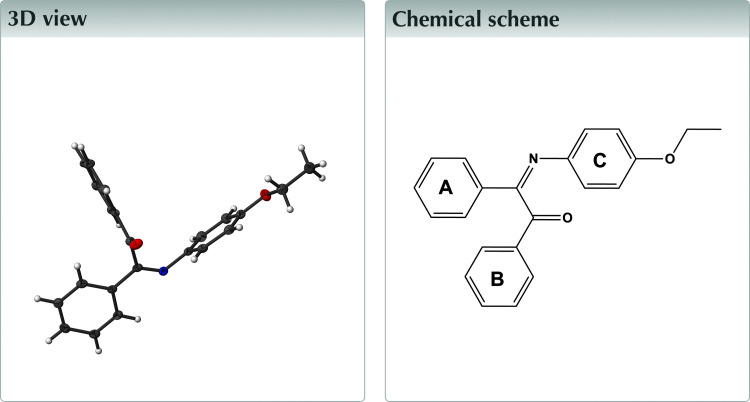

## Structure description

**Figure 1:**
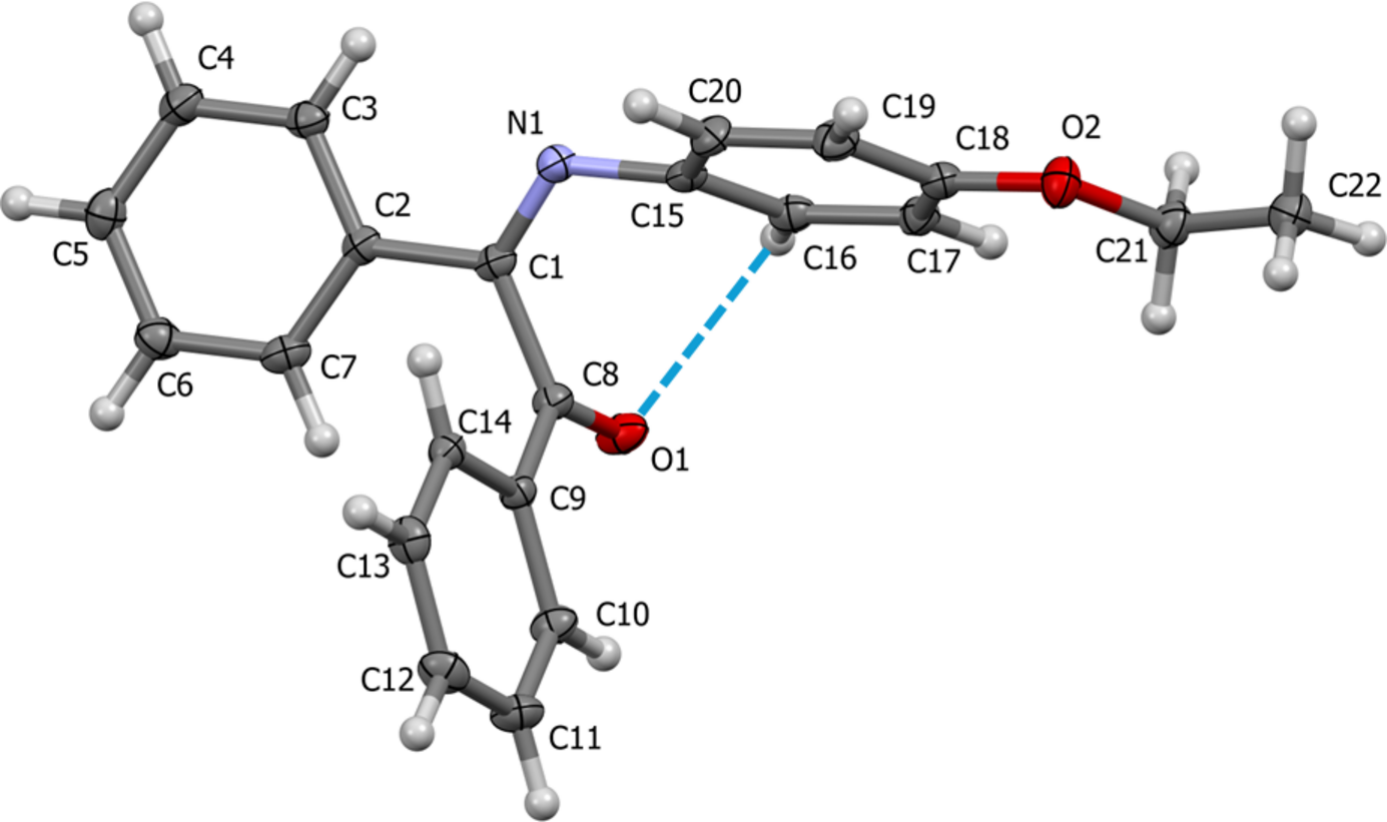
A view of the mol­ecular structure of (**I**) with displacement ellipsoids drawn at the 50% probability level. The intra­molecular C—H⋯O hydrogen bond (Table 1 ▸) is shown as a dashed blue line.

As part of our ongoing studies of Schiff bases (Ziani *et al.*, 2023 ▸; Bouchama *et al.*, 2007 ▸), we describe herein the synthesis and crystal structure of the title imino-ketone compound, (**I**). The mol­ecular structure of (**I**) is illustrated in Fig. 1 ▸. The configuration about the azomethine bond, C1=N1, is *E*, with a bond length of 1.278 (2) Å. This value is essentially identical to that observed for the 4-meth­oxy­phenyl analogue (**II**), *viz*. 1.277 (2) Å (Wang *et al.*, 2021 ▸). In the 1,2-di­phenyl­ethan-1-one unit, the aromatic rings *A* (C2–C7) and *B* (C9–C14) are inclined to each other by 80.43 (7)°. The 4-eth­oxy­phenyl ring *C* (C15–C20) is inclined to rings *A* and *B* by 68.56 (7) and 81.91 (7)°, respectively. In the 4-meth­oxy­phenyl analogue (**II**), these dihedral angles are similar; *A*/*B* is 79.85 (6)°, *A*/*C* is 70.06 (6)° and *B*/*C* is 77.26 (6)°. The 4-eth­oxy­phenyl moiety is almost co-planar with the eth­oxy unit (O2/C21/C22), being inclined to aromatic ring *C* by only 1.33 (15)°. There is a short intra­molecular C16—H16⋯O1 contact in the mol­ecule (Fig. 1 ▸ and Table 1 ▸).

**Figure 2:**
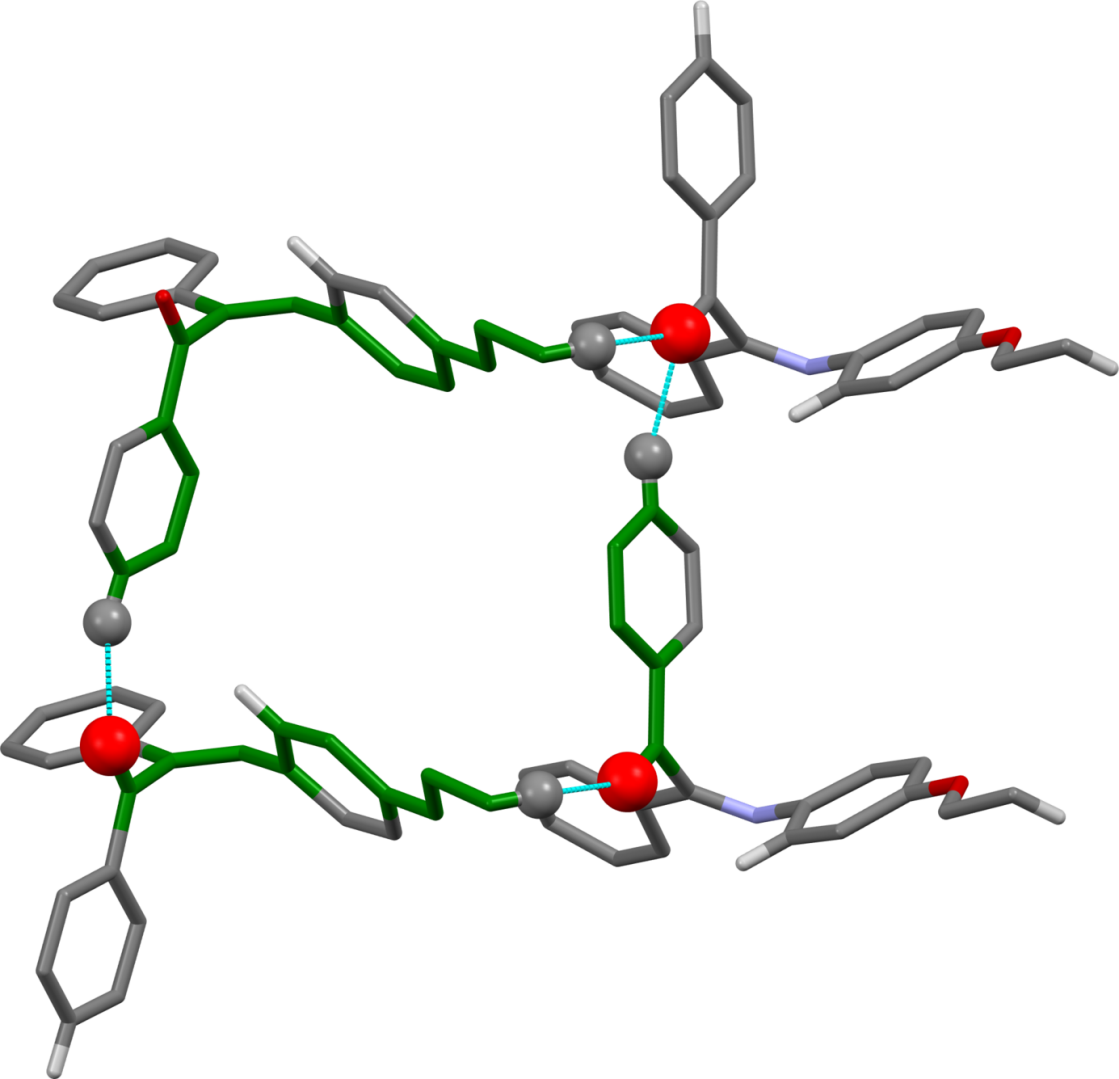
A view of four symmetry-related mol­ecules of (**I**) that are linked by C—H⋯O hydrogen bonds (Table 1 ▸) to form a square-like unit with an 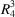
(36) ring motif. (The acceptor O atoms are red, the donor H atoms are grey and the ring atoms are green.)

**Figure 3:**
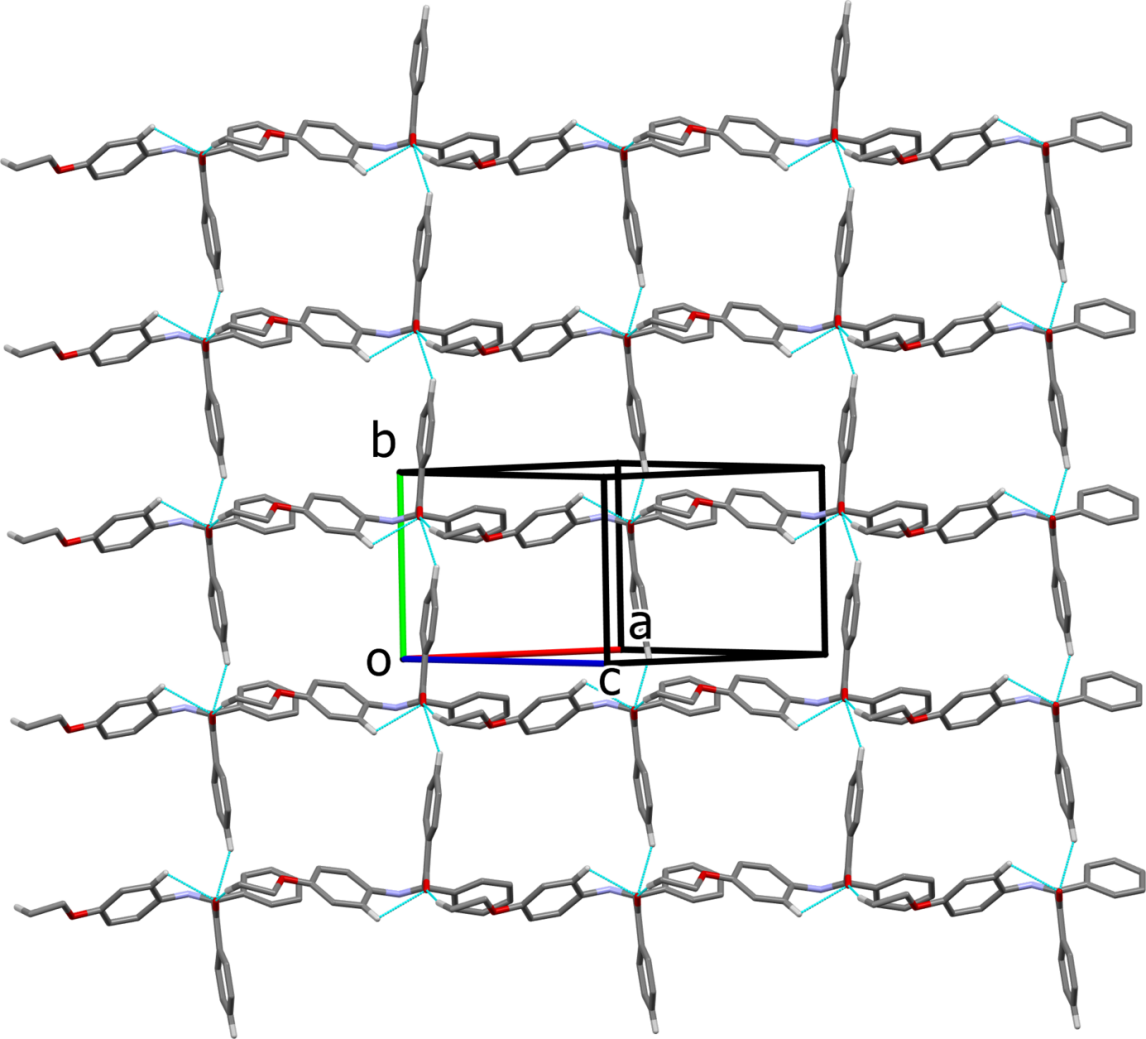
A view of the sheet-like hydrogen-bonded arrangement of symmetry-related mol­ecules in the crystal of (**I**), lying parallel to the (10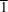) plane.

In the crystal, four symmetry-related mol­ecules are linked by C—H⋯O hydrogen bonds, forming a square-like unit with an 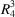
(36) ring motif (Table 1 ▸ and Fig. 2 ▸). These units are linked by bifurcated C—H⋯O hydrogen bonds, forming sheets lying parallel to the (10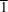) plane (Fig. 3 ▸).

## Synthesis and crystallization

Equimolar amounts of benzil (0.210 g, 0.01 mmol) and 4-eth­oxy­aniline (0.137 g, 0.01 mmol) were mixed in absolute ethanol (30 ml) and refluxed under stirring for 3 h. After cooling to room temperature, the solvent was allowed to evaporate slowly, yielding yellow needle-like crystals (yield 0.249 g, 76%, m.p. 529.5 K).

## Refinement

Crystal data, data collection and structure refinement details are summarized in Table 2 ▸.

## Supplementary Material

Crystal structure: contains datablock(s) global, I. DOI: 10.1107/S2414314626007406/zl4101sup1.cif

Structure factors: contains datablock(s) I. DOI: 10.1107/S2414314626007406/zl4101Isup2.hkl

CCDC reference: 2574557

Additional supporting information:  crystallographic information; 3D view; checkCIF report

## Figures and Tables

**Table 1:** Hydrogen-bond geometry (Å, °)

*D*—H⋯*A*	*D*—H	H⋯*A*	*D*⋯*A*	*D*—H⋯*A*
C16—H16⋯O1	0.95	2.56	3.068 (2)	114
C12—H12⋯O1^i^	0.95	2.41	3.279 (2)	151
C22—H22*A*⋯O1^ii^	0.98	2.55	3.462 (2)	155

Symmetry codes: (i) 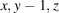; (ii) 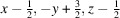.

**Table 2:** Experimental details

Crystal data	
Chemical formula	C_22_H_19_NO_2_
*M*_r_	329.38
Crystal system, space group	Monoclinic, *P*2_1_/*n*
Temperature (K)	100
*a*, *b*, *c* (Å)	13.0674 (4), 8.0633 (2), 16.4309 (5)
β (°)	101.412 (3)
*V* (Å^3^)	1697.04 (9)
*Z*	4
Radiation type	Mo *K*α
μ (mm^−1^)	0.08
Crystal size (mm)	0.32 × 0.17 × 0.05

Data collection	
Diffractometer	Xcalibur, Eos, Nova
Absorption correction	Multi-scan (*CrysAlis PRO*; Rigaku OD, 2022 ▸)
*T*_min_, *T*_max_	0.568, 1.000
No. of measured, independent and observed [*I* > 2σ(*I*)] reflections	22651, 4240, 3037
*R*_int_	0.050
(sin θ/λ)_max_ (Å^−1^)	0.691

Refinement	
*R*[*F*^2^ > 2σ(*F*^2^)], *wR*(*F*^2^), *S*	0.052, 0.117, 1.04
No. of reflections	4240
No. of parameters	227
H-atom treatment	H-atom parameters constrained
Δρ_max_, Δρ_min_ (e Å^−3^)	0.27, −0.26

Computer programs: *CrysAlis PRO* (Rigaku OD, 2022 ▸), *SHELXT2018/2* (Sheldrick, 2015*a* ▸), *SHELXL2025/1* (Sheldrick, 2015*b* ▸), *Mercury* (Macrae *et al.*, 2020 ▸), *OLEX2* (Dolomanov *et al.*, 2009 ▸), *PLATON* (Spek, 2020 ▸) and *publCIF* (Westrip, 2010 ▸).

## full crystallographic data

### (2E)-2-[(4-Ethoxyphenyl)imino]-1,2-diphenylethanone Crystal data

**Table:**

C_22_H_19_NO_2_	*F*(000) = 696
*M**_r_* = 329.38	*D*_x_ = 1.289 Mg m^−^^3^
Monoclinic, *P*2_1_/*n*	Mo *K*α radiation, λ = 0.71073 Å
*a* = 13.0674 (4) Å	Cell parameters from 7263 reflections
*b* = 8.0633 (2) Å	θ = 4.0–28.9°
*c* = 16.4309 (5) Å	µ = 0.08 mm^−^^1^
β = 101.412 (3)°	*T* = 100 K
*V* = 1697.04 (9) Å^3^	Plate, yellow
*Z* = 4	0.32 × 0.17 × 0.05 mm

### (2E)-2-[(4-Ethoxyphenyl)imino]-1,2-diphenylethanone Data collection

**Table:**

Xcalibur, Eos, Nova diffractometer	3037 reflections with *I* > 2σ(*I*)
Detector resolution: 15.9897 pixels mm^-1^	*R*_int_ = 0.050
ω scans	θ_max_ = 29.4°, θ_min_ = 3.4°
Absorption correction: multi-scan (CrysAlisPro; Rigaku OD, 2022)	*h* = −17→17
*T*_min_ = 0.568, *T*_max_ = 1.000	*k* = −11→10
22651 measured reflections	*l* = −22→22
4240 independent reflections	

### (2E)-2-[(4-Ethoxyphenyl)imino]-1,2-diphenylethanone Refinement

**Table:**

Refinement on *F*^2^	Primary atom site location: dual
Least-squares matrix: full	Secondary atom site location: difference Fourier map
*R*[*F*^2^ > 2σ(*F*^2^)] = 0.052	Hydrogen site location: inferred from neighbouring sites
*wR*(*F*^2^) = 0.117	H-atom parameters constrained
*S* = 1.04	*w* = 1/[σ^2^(*F*_o_^2^) + (0.0451*P*)^2^ + 0.5117*P*] where *P* = (*F*_o_^2^ + 2*F*_c_^2^)/3
4240 reflections	(Δ/σ)_max_ < 0.001
227 parameters	Δρ_max_ = 0.27 e Å^−^^3^
0 restraints	Δρ_min_ = −0.26 e Å^−^^3^

### (2E)-2-[(4-Ethoxyphenyl)imino]-1,2-diphenylethanone Special details

**Table:**

Geometry. All esds (except the esd in the dihedral angle between two l.s. planes) are estimated using the full covariance matrix. The cell esds are taken into account individually in the estimation of esds in distances, angles and torsion angles; correlations between esds in cell parameters are only used when they are defined by crystal symmetry. An approximate (isotropic) treatment of cell esds is used for estimating esds involving l.s. planes.
Refinement. The hydrogen atoms were fixed geometrically (C—H = 0.95–0.99 Å) and allowed to ride on their parent atoms with *U*_iso_(H) = 1.5*U*_eq_(*C*-methyl) and 1.2*U*_eq_(C) for other H atoms.

### (2E)-2-[(4-Ethoxyphenyl)imino]-1,2-diphenylethanone Fractional atomic coordinates and isotropic or equivalent isotropic displacement parameters (Å^2^)

**Table:**

	*x*	*y*	*z*	*U*_iso_*/*U*_eq_	
O1	0.37863 (9)	0.72732 (14)	0.72318 (7)	0.0230 (3)	
O2	−0.00143 (8)	0.65400 (14)	0.42270 (7)	0.0233 (3)	
N1	0.42408 (10)	0.75710 (16)	0.54025 (8)	0.0177 (3)	
C1	0.47276 (12)	0.72080 (18)	0.61342 (9)	0.0159 (3)	
C2	0.58625 (11)	0.75382 (18)	0.63777 (9)	0.0150 (3)	
C3	0.63793 (12)	0.84505 (19)	0.58563 (9)	0.0187 (3)	
H3	0.599688	0.888983	0.535014	0.022*	
C4	0.74416 (12)	0.8714 (2)	0.60744 (10)	0.0210 (3)	
H4	0.778474	0.934724	0.572067	0.025*	
C5	0.80129 (12)	0.8062 (2)	0.68071 (10)	0.0220 (4)	
H5	0.874683	0.822321	0.694735	0.026*	
C6	0.75096 (12)	0.7176 (2)	0.73313 (10)	0.0221 (4)	
H6	0.789725	0.673329	0.783465	0.026*	
C7	0.64419 (12)	0.6935 (2)	0.71241 (10)	0.0208 (4)	
H7	0.609858	0.634988	0.749469	0.025*	
C8	0.42051 (11)	0.64005 (19)	0.67898 (9)	0.0164 (3)	
C9	0.42599 (11)	0.45736 (18)	0.68773 (9)	0.0150 (3)	
C10	0.39198 (12)	0.3842 (2)	0.75483 (9)	0.0195 (3)	
H10	0.362780	0.450853	0.792138	0.023*	
C11	0.40093 (13)	0.2151 (2)	0.76670 (10)	0.0232 (4)	
H11	0.377345	0.165044	0.812001	0.028*	
C12	0.44422 (12)	0.1181 (2)	0.71271 (10)	0.0229 (4)	
H12	0.451685	0.002030	0.721944	0.027*	
C13	0.47678 (12)	0.1891 (2)	0.64523 (10)	0.0199 (3)	
H13	0.505542	0.121833	0.607927	0.024*	
C14	0.46712 (11)	0.35854 (19)	0.63259 (9)	0.0166 (3)	
H14	0.488628	0.407542	0.586138	0.020*	
C15	0.31464 (12)	0.73071 (19)	0.51553 (9)	0.0175 (3)	
C16	0.24190 (12)	0.80883 (19)	0.55370 (9)	0.0189 (3)	
H16	0.265110	0.877720	0.600625	0.023*	
C17	0.13555 (12)	0.78732 (19)	0.52400 (10)	0.0195 (3)	
H17	0.086599	0.841725	0.550558	0.023*	
C18	0.10068 (12)	0.6867 (2)	0.45574 (9)	0.0188 (3)	
C19	0.17346 (12)	0.6111 (2)	0.41623 (10)	0.0212 (4)	
H19	0.150243	0.543058	0.368988	0.025*	
C20	0.27838 (12)	0.6342 (2)	0.44505 (9)	0.0213 (4)	
H20	0.327052	0.583954	0.416726	0.026*	
C21	−0.07850 (12)	0.7274 (2)	0.46183 (11)	0.0232 (4)	
H21A	−0.071613	0.849674	0.462343	0.028*	
H21B	−0.069898	0.688132	0.519880	0.028*	
C22	−0.18352 (12)	0.6770 (2)	0.41299 (10)	0.0250 (4)	
H22A	−0.189758	0.711703	0.355052	0.038*	
H22B	−0.238460	0.730179	0.436621	0.038*	
H22C	−0.190857	0.556242	0.415420	0.038*	

### (2E)-2-[(4-Ethoxyphenyl)imino]-1,2-diphenylethanone Atomic displacement parameters (Å^2^)

**Table:**

	*U*^11^	*U*^22^	*U*^33^	*U*^12^	*U*^13^	*U*^23^
O1	0.0319 (7)	0.0175 (6)	0.0236 (6)	0.0029 (5)	0.0153 (5)	−0.0003 (5)
O2	0.0178 (6)	0.0292 (7)	0.0237 (6)	0.0011 (5)	0.0058 (5)	−0.0046 (5)
N1	0.0174 (7)	0.0182 (7)	0.0183 (6)	0.0001 (5)	0.0057 (5)	0.0024 (5)
C1	0.0205 (8)	0.0118 (7)	0.0174 (7)	0.0008 (6)	0.0086 (6)	−0.0011 (6)
C2	0.0179 (8)	0.0110 (7)	0.0177 (7)	0.0005 (6)	0.0072 (6)	−0.0023 (6)
C3	0.0223 (8)	0.0173 (8)	0.0178 (7)	0.0014 (6)	0.0073 (6)	0.0013 (6)
C4	0.0226 (8)	0.0185 (8)	0.0244 (8)	−0.0015 (7)	0.0109 (7)	0.0000 (7)
C5	0.0188 (8)	0.0201 (8)	0.0273 (9)	−0.0017 (7)	0.0052 (7)	−0.0045 (7)
C6	0.0229 (9)	0.0219 (9)	0.0201 (8)	−0.0017 (7)	0.0011 (7)	0.0012 (7)
C7	0.0258 (9)	0.0189 (8)	0.0191 (8)	−0.0042 (7)	0.0079 (7)	0.0007 (6)
C8	0.0173 (8)	0.0167 (8)	0.0158 (7)	−0.0006 (6)	0.0049 (6)	0.0003 (6)
C9	0.0138 (7)	0.0142 (7)	0.0169 (7)	−0.0012 (6)	0.0029 (6)	0.0008 (6)
C10	0.0235 (8)	0.0184 (8)	0.0183 (8)	−0.0014 (7)	0.0081 (6)	−0.0015 (6)
C11	0.0289 (9)	0.0210 (9)	0.0210 (8)	−0.0048 (7)	0.0078 (7)	0.0033 (7)
C12	0.0283 (9)	0.0135 (8)	0.0261 (8)	−0.0011 (7)	0.0037 (7)	0.0008 (7)
C13	0.0207 (8)	0.0172 (8)	0.0215 (8)	0.0017 (6)	0.0034 (6)	−0.0043 (7)
C14	0.0156 (7)	0.0185 (8)	0.0162 (7)	−0.0013 (6)	0.0046 (6)	−0.0002 (6)
C15	0.0180 (8)	0.0180 (8)	0.0178 (7)	−0.0004 (6)	0.0066 (6)	0.0054 (6)
C16	0.0233 (8)	0.0156 (8)	0.0185 (8)	−0.0015 (6)	0.0060 (6)	0.0007 (6)
C17	0.0211 (8)	0.0179 (8)	0.0217 (8)	0.0029 (6)	0.0097 (6)	0.0022 (7)
C18	0.0183 (8)	0.0199 (8)	0.0189 (8)	0.0000 (6)	0.0056 (6)	0.0042 (6)
C19	0.0233 (8)	0.0241 (9)	0.0169 (7)	−0.0003 (7)	0.0058 (6)	−0.0018 (7)
C20	0.0215 (8)	0.0259 (9)	0.0187 (8)	0.0029 (7)	0.0094 (6)	0.0010 (7)
C21	0.0213 (8)	0.0228 (9)	0.0277 (9)	0.0025 (7)	0.0103 (7)	−0.0003 (7)
C22	0.0216 (9)	0.0285 (9)	0.0262 (9)	0.0027 (7)	0.0078 (7)	0.0021 (7)

### (2E)-2-[(4-Ethoxyphenyl)imino]-1,2-diphenylethanone Geometric parameters (Å, º)

**Table:**

O1—C8	1.2162 (18)	C11—H11	0.9500
O2—C18	1.3630 (19)	C11—C12	1.385 (2)
O2—C21	1.4263 (18)	C12—H12	0.9500
N1—C1	1.2779 (19)	C12—C13	1.387 (2)
N1—C15	1.423 (2)	C13—H13	0.9500
C1—C2	1.482 (2)	C13—C14	1.384 (2)
C1—C8	1.530 (2)	C14—H14	0.9500
C2—C3	1.400 (2)	C15—C16	1.389 (2)
C2—C7	1.395 (2)	C15—C20	1.398 (2)
C3—H3	0.9500	C16—H16	0.9500
C3—C4	1.380 (2)	C16—C17	1.390 (2)
C4—H4	0.9500	C17—H17	0.9500
C4—C5	1.388 (2)	C17—C18	1.386 (2)
C5—H5	0.9500	C18—C19	1.394 (2)
C5—C6	1.381 (2)	C19—H19	0.9500
C6—H6	0.9500	C19—C20	1.372 (2)
C6—C7	1.383 (2)	C20—H20	0.9500
C7—H7	0.9500	C21—H21A	0.9900
C8—C9	1.481 (2)	C21—H21B	0.9900
C9—C10	1.398 (2)	C21—C22	1.502 (2)
C9—C14	1.392 (2)	C22—H22A	0.9800
C10—H10	0.9500	C22—H22B	0.9800
C10—C11	1.380 (2)	C22—H22C	0.9800
			
C18—O2—C21	117.58 (12)	C13—C12—H12	119.7
C1—N1—C15	121.28 (13)	C12—C13—H13	120.2
N1—C1—C2	119.89 (13)	C14—C13—C12	119.62 (14)
N1—C1—C8	123.46 (13)	C14—C13—H13	120.2
C2—C1—C8	116.64 (13)	C9—C14—H14	119.9
C3—C2—C1	120.48 (13)	C13—C14—C9	120.19 (14)
C7—C2—C1	120.99 (13)	C13—C14—H14	119.9
C7—C2—C3	118.53 (14)	C16—C15—N1	122.83 (14)
C2—C3—H3	119.9	C16—C15—C20	118.45 (14)
C4—C3—C2	120.29 (14)	C20—C15—N1	118.45 (14)
C4—C3—H3	119.9	C15—C16—H16	119.6
C3—C4—H4	119.8	C15—C16—C17	120.73 (15)
C3—C4—C5	120.49 (15)	C17—C16—H16	119.6
C5—C4—H4	119.8	C16—C17—H17	119.9
C4—C5—H5	120.1	C18—C17—C16	120.20 (14)
C6—C5—C4	119.73 (15)	C18—C17—H17	119.9
C6—C5—H5	120.1	O2—C18—C17	125.09 (14)
C5—C6—H6	120.0	O2—C18—C19	115.73 (14)
C5—C6—C7	120.06 (15)	C17—C18—C19	119.18 (14)
C7—C6—H6	120.0	C18—C19—H19	119.8
C2—C7—H7	119.6	C20—C19—C18	120.49 (15)
C6—C7—C2	120.85 (15)	C20—C19—H19	119.8
C6—C7—H7	119.6	C15—C20—H20	119.6
O1—C8—C1	119.36 (14)	C19—C20—C15	120.89 (15)
O1—C8—C9	122.45 (13)	C19—C20—H20	119.6
C9—C8—C1	118.16 (12)	O2—C21—H21A	110.2
C10—C9—C8	118.64 (13)	O2—C21—H21B	110.2
C14—C9—C8	121.59 (13)	O2—C21—C22	107.39 (13)
C14—C9—C10	119.73 (14)	H21A—C21—H21B	108.5
C9—C10—H10	120.1	C22—C21—H21A	110.2
C11—C10—C9	119.81 (14)	C22—C21—H21B	110.2
C11—C10—H10	120.1	C21—C22—H22A	109.5
C10—C11—H11	119.9	C21—C22—H22B	109.5
C10—C11—C12	120.12 (15)	C21—C22—H22C	109.5
C12—C11—H11	119.9	H22A—C22—H22B	109.5
C11—C12—H12	119.7	H22A—C22—H22C	109.5
C11—C12—C13	120.51 (15)	H22B—C22—H22C	109.5
			
O1—C8—C9—C10	−7.1 (2)	C8—C1—C2—C3	−172.28 (13)
O1—C8—C9—C14	175.02 (14)	C8—C1—C2—C7	8.5 (2)
O2—C18—C19—C20	−179.23 (14)	C8—C9—C10—C11	−176.86 (14)
N1—C1—C2—C3	8.9 (2)	C8—C9—C14—C13	176.21 (14)
N1—C1—C2—C7	−170.37 (14)	C9—C10—C11—C12	0.5 (2)
N1—C1—C8—O1	−86.83 (19)	C10—C9—C14—C13	−1.6 (2)
N1—C1—C8—C9	95.03 (18)	C10—C11—C12—C13	−1.5 (2)
N1—C15—C16—C17	175.87 (14)	C11—C12—C13—C14	0.9 (2)
N1—C15—C20—C19	−176.97 (14)	C12—C13—C14—C9	0.7 (2)
C1—N1—C15—C16	61.3 (2)	C14—C9—C10—C11	1.0 (2)
C1—N1—C15—C20	−124.78 (16)	C15—N1—C1—C2	−178.45 (13)
C1—C2—C3—C4	−178.13 (14)	C15—N1—C1—C8	2.8 (2)
C1—C2—C7—C6	176.87 (14)	C15—C16—C17—C18	0.2 (2)
C1—C8—C9—C10	170.95 (13)	C16—C15—C20—C19	−2.8 (2)
C1—C8—C9—C14	−6.9 (2)	C16—C17—C18—O2	178.41 (14)
C2—C1—C8—O1	94.38 (17)	C16—C17—C18—C19	−1.6 (2)
C2—C1—C8—C9	−83.76 (17)	C17—C18—C19—C20	0.8 (2)
C2—C3—C4—C5	0.8 (2)	C18—O2—C21—C22	178.44 (13)
C3—C2—C7—C6	−2.4 (2)	C18—C19—C20—C15	1.4 (2)
C3—C4—C5—C6	−1.6 (2)	C20—C15—C16—C17	2.0 (2)
C4—C5—C6—C7	0.4 (2)	C21—O2—C18—C17	−0.7 (2)
C5—C6—C7—C2	1.7 (2)	C21—O2—C18—C19	179.29 (14)
C7—C2—C3—C4	1.1 (2)		

### (2E)-2-[(4-Ethoxyphenyl)imino]-1,2-diphenylethanone Hydrogen-bond geometry (Å, º)

**Table:**

*D*—H···*A*	*D*—H	H···*A*	*D*···*A*	*D*—H···*A*
C16—H16···O1	0.95	2.56	3.068 (2)	114
C12—H12···O1^i^	0.95	2.41	3.279 (2)	151
C22—H22*A*···O1^ii^	0.98	2.55	3.462 (2)	155

Symmetry codes: (i) *x*, *y*−1, *z*; (ii) *x*−1/2, −*y*+3/2, *z*−1/2.
